# Social workers’ perspectives on a medical home model for children and adolescents in out of home care – an interview study

**DOI:** 10.1186/s12913-021-06737-1

**Published:** 2021-08-12

**Authors:** Nina Johansson, Karin Fängström, Georgina Warner

**Affiliations:** Child Health and Parenting (CHAP), Department of Public Health and Caring Sciences, Uppsala University, Husargatan 3, 751 22 Uppsala, Sweden

**Keywords:** Children and adolescents, Health, Out of home care, Medical home model, Social services

## Abstract

**Background:**

This study seeks to explore how social workers have perceived and experienced a medical home model for children and adolescents in out-of-home care in Uppsala County, Sweden.

**Method:**

A qualitative explorative study was conducted, which involved ten semi-structured individual telephone interviews with social workers. The study sample included employees within the social service, working in a specialised case unit who had experience of referring children and/or adolescents to the medical home model called Hälsofam. Data were analysed inductively using thematic analysis.

**Results:**

The findings of the current study indicated that working with Hälsofam has offered social workers a way into the health care sector and an active collaborative working situation, with focus on organised work across the ‘silos’ of care services. However, the findings raised the question of whether or not *all* children and adolescents have the same possibility to receive care from Hälsofam.

**Conclusion:**

The findings indicated that the Hälsofam model had a positive impact on the interrelations between the social service and the health care sector. Yet, findings showed that personal views of the social worker and the societal situation in which they operate create limitations for providing care for every child and adolescent. This study adds to the extant literature for it addresses the limitations within the work of children and adolescents in out-of-home care.

**Supplementary Information:**

The online version contains supplementary material available at 10.1186/s12913-021-06737-1.

## Background

In Sweden, outcomes for children in out-of-home care (OHC) have been disappointing [1]. They are exposed to a greater risk for somatic health problems [2], are less likely to be up-to-date with their immunisation [3, 4] and have a higher risk of obesity, overweight and developmental delays [5]. Children and adolescents in care have to a higher extent diagnosable mental health disorders [6–10]. A well-functioning system of health assessments and examinations in the process for OHC placements is essential to discover potential health issues. Today in Sweden, basic preventive needs are unmet and chronic conditions unidentified. There is a need for a comprehensive and continuous system.

In 2018, Swedish statistics showed that 38,800 children and adolescents were placed in care by The National Board of Health and Welfare [11]. ‘Placed in care’ refers to social out-of-home care (OHC), which includes foster care, residential care and secure units [12]. Children and adolescents can be placed in OHC in accordance to either The Swedish Social Services Act SFS, 2001:453 [13] or The Swedish Care of Young Persons (Special Provisions) Act SFS, 1990:52 [14]. To place a child or adolescent in OHC according to Act SFS, 2001:453 [13], the social service is obliged to have an active investigation within the family and a consensual agreement between with all concerning parties. Being placed in care according to Act SFS, 2001:453 is defined as voluntary care. Children and adolescents who receive care according to SFS, 1990:52 [14] are in an acute state, which requires an immediate placement in OHC and no consensual agreement. This is defined as compulsory care. In 2018, 77% of the OHC placements were voluntary care and 23% were compulsory care [11].

In accordance with The Swedish Local Government Act SFS; 2017:725 [15], local authorities and regions are self-governed. On the basis of the municipal autonomy, local authorities and regions operate according to specified laws or regulations. Swedish Local Authorities are responsible, according to SFS, 2001:453 [13], for the social services within their area. Local authorities and regions in Sweden have agreements for coordinating health care and social services for children and adolescents in the process of OHC placements [16]. The agreements cover somatic, mental and dental health. Nationally, regulations specifies that children and adolescents’ in OHC should have a care plan with follow-up objectives for health care needs [17]. Regulations and general advice specifying what should be included in the health assessments was initiated nationally from 1st January 2020 [18].

To date, research on health assessments of children and adolescents in OHC shows a low percentage of referrals [19]. In national surveys made by The Swedish Association of Local Authorities and Regions [16, 20], the findings of 2014 and 2016 shows that children and adolescents born in Sweden and placed in *compulsory* care received health assessments to a higher extent than children and adolescents placed in *voluntary* care.

Medical home models are widely supported [21]. In 2007, the model was endorsed by a number of professional bodies, including the American Academy of Family Physicians, the American College of Physicians, the American Academy of Paediatrics, and the American Osteopathic Association. The model aims to strengthen the process of providing comprehensive primary care and to improve primary health care organisation and conditions by emphasising care coordination. A medical home model for children should be coordinated with multiple sectors including health care providers and relevant community actors. Despite the popularity of the model, a recent systematic review demonstrated there are very few previous studies that show significant impact of organisational models within the field of health and dental care for children and adolescents in OHC. There is a fundamental need for rigorous evaluations of current models [22]. However, one can find several narrative overviews of organisational models. From the UK, three research papers provide narrative overviews on various models within the field of mental health care for children and adolescents in OHC [23–25], and there is a further example on the care and health needs of children in residential care the islands of Malta and Gozo [26].

In an evaluation of the societal costs of organisational models for health care of children and young people in OHC [22], the initial cost to develop a model was calculated as 5,5 million Swedish Krona, with a yearly cost of 3300 per child. With this kind of organisational model, the expectation is that children’s consumption of health care and dental care would increase. Although this also has a cost associated, it would positively contribute to the health of children and adolescents in OHC, which is not only desirable from an ethical, legal and social aspect but could also prevent more serious health issues that would require greater service consumption later on [22].

This qualitative research study seeks to address the need for further studies on the topic.

### The Hälsofam model

Hälsofam is a medical home model for children, 0–17 years old, investigated by the Child Protection Services (CPS) and provides multi-professional coordinated consultation and care [27]. The focus of the model is to strengthen the children and adolescents’ resilience, to deliver trauma-aware care, to increase the parental support and the participation of the youth. This is made by a Child Protection Team (CPT) at Uppsala University Hospital.

The Hälsofam user group is children, 0–17 years, living in or near Uppsala region. In 2019, the population of children in the age between 0 and 19 were 522,33 out of the total population of 230,767 [28]. In the latest statistics from 2016, 7 out of 1000 children were placed in OHC care in Uppsala region [29]. Reasons for being placed in OHC can be due to parental limitation in providing sufficient care, parents not being suitable for being a caregiver or that the behaviour of the child or adolescent can be a risk to themselves or others [30]. Children and adolescents who come to Hälsofam often have experiences of maltreatment and/or violence, and have an increased need for health, dental and medical care, given that they often have missed out on routine health checks at well-baby clinics, school health care and dental check-ups.

In a case review from 2020, thirty randomly selected Hälsofam Electronic Medical Records (EMR) of children aged 0–17 years from April 2016 to April 2019, were studied [30]. The aim was to study EMR to assess the morbidity among children and adolescents in OHC. The medical records showed that the youngest to be referred to Hälsofam was 2 months old, and 3 out of 10 children were less than 1 year old at their first Hälsofam visit. At least 5 out of 10 children had more than one reason for being place in OHC and in the group aged 13–17, only one child had a parent present at their first visit. Research findings concerning the parental role showed that 6 children had at least one parent who has been sentenced to prison, 7 children had at least one parent with active substance abuse and 13 children had at least one parent with a psychiatric diagnosis. Other findings were that 7 out of 10 children had high absences from school, at least 5 out of 10 children experienced bullying in school and that 9 out of 10 children had sleep problems. The study concluded that that children and adolescents who come to the Hälsofam service have an overrepresentation in morbidity, which requires referrals for health examinations and follow-ups.

The Hälsofam process commences with a multidisciplinary assessment taking place when a child enters the service. This includes a physical exam, a developmental assessment by a paediatrician, a psychological assessment by a child psychologist and, if required, referrals to associated health services. The assessment concludes in a health report delivered to CPS. The model includes, on average, two follow up visits in the first year. The child is followed up even if the current OHC placement is in another region. Follow up with the birth family is offered if the child exits care or the court does not approve CPS placement. The follow up is tailored to the child’s individual needs. A handover takes place when the form of care changes. Hälsofam personnel coordinate with CPS regarding the child’s health plan and match with placement and (proposed) multi agency quality assurance at the child level.

Although there is an acknowledged lack of empirical research on organisational models within the field of health and dental care for children and adolescents in OHC, a body of literature exists on multidisciplinary team responses to child abuse. A recent scoping review [31] of cross-agency collaboration in child abuse cases highlighted key factors that may influence the quality of multidisciplinary working models. The most commonly cited factor was the need to unite the various roles and responsibilities of workers [31]. Clearly articulated procedures, cross-agency training, and communication and information sharing practices were all identified as important [31]. Whilst this literature can give insight to how the quality of medical home models such as Hälsofam can be improved, dedicated evaluation of the model is warranted. Organisations are complex, adaptive systems and organisational developments require dedicated evaluation to understand what works and what does not in particular circumstances [32, 33]. A number of benefits of organisational development evaluation have been identified [32]. Particularly relevant to the current phase of Hälsofam in the Uppsala region, evaluation can help to: understand how the model can be developed or improved, as well as enhance relationships and energise practitioners [32].

As key stakeholders in the model, there is a need for clarification around how social workers perceive Hälsofam. Previous research indicates that social work activity is influenced by role expectation [34]. The Hälsofam process is reliant on social workers actively participating. The social workers’ expectations should be aligned with the logic model of Hälsofam to enable the desired outcomes to be achieved [35]. Thus the goal of the present study was to answer the following question: How do social workers perceive the Hälsofam model? More specifically, we aimed to address the following research questions: How do individual social workers perceive Hälsofam? How is Hälsofam perceived within the social services more broadly? How does Hälsofam interact with policy expectations placed up social workers? How is the interrelation between social and health services experienced? The findings from this study could fill a current knowledge gap regarding medical care for children in OHC in Sweden.

## Theoretical framework

### The ecological theory

**Fig. 1:**
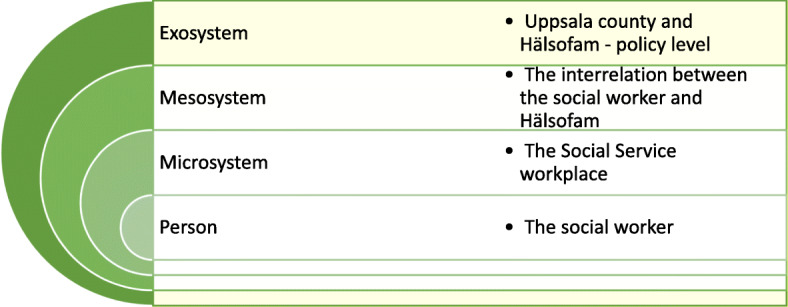
An ecological system model of social work. Based on “The Ecology of Human Development: Experiments by Nature and Design.” by U. Bronfenbrenner, 1981, Harvard University Press

The ecological theory [36], emphasises the need for studying the context in which people live and behave. The environment where a developing individual interacts is seen as a set of ecological structures, see Fig. 1. Bronfenbrenner defines the innermost level, the *system of the person*, as the immediate setting for an individual. The *microsystem* is described as a set of activities, functions and interpersonal relations perceived by the individual in a setting. In the current research it was used to study the setting for social workers and the interactions, activities and roles that they experience at their workplace, working with Hälsofam. The *mesosystem* refers to the interrelation between two or several settings in which the individuals have active roles. For this study, it was used to consider the interrelation between the Hälsofam personnel and the staff at the social service. The *exosystem* refers to one or several settings that affects or are affected by a setting that do not involve the individual as an active actor. It was used to study how the social workers were affected by the setting of the Uppsala County and Hälsofam including, but not exclusive to, policies adopted by the organisations.

## Methods

### Setting

Hälsofam is currently delivered by the Child Protection Team at Uppsala University Hospital. Uppsala University Hospital is one of the seven University hospital in Sweden. The service gives priority to the eight municipalities within the region but are available for nearby municipalities. In Uppsala County, the eight municipalities are themselves responsible for the social service within their area.

The Hälsofam user group is children, 0–17 years, living in or near Uppsala region. In 2019, the population of children in the age between 0 and 19 years was 52,233 out of the total population of 230,767 [28]. In the latest statistics from 2016, 7 out of a 1000 children were placed in OHC care in Uppsala region [29].

### Recruitment strategy

The study population consisted of social workers. A mix of purposeful sampling and snowballing sample technique was used to collect participants. The criteria of inclusion in the study sample were:

i. Employees within the social service
ii. Social workers, working in a specialised case unit with long-term care
iii. Experience and knowledge about referring children and/or adolescents to Hälsofam

In terms of sample size, the target was to reach an initial analysis sample of 10 social workers. If no new information had emerged from the last 3 conducted interviews, then recruitment would stop. However, if new ideas were emerging then more social workers would be recruited. This would continue until no new ideas were raised. This was defined as having a stopping criterion of 3 [37]. Within this process, when no new information had emerged after the last 3 interviews, the research was considered as having data saturation.

Contact was established with supervisors and social service personnel from March to May 2020. The process began, by guidance of Patton [38], by asking well-situated personnel to locate information-rich key informants. By asking representatives at Hälsofam at the Uppsala University Children’s Hospital, a chain of recommended informants was created. On the initial list of information-rich key informants, there were 11 potential candidates. The responsible supervisors were then contacted by email with information about the research and with a request to approve their employees being approached. With a given approval, the employees were then approached by the responsible researcher with information about the research and asked for their interest in participating. The responsible researcher was then responsive to the respondent’s suggestions for scheduled interviews. After the conducted telephone interview, the interviewer requested recommendations for other potential participants. In the event of a given recommendation, the same procedure was performed. Out of the 11 that was on the initial list, 7 took part in an interview. Within the process of recruiting participants, 4 recommendations were given. One supervisor did not give approval to contact their employee, with the given reason of too high workload at the time. Given reasons not to participate were too high workload, not having enough experience of the model or that the person simply stopped answering emails and phone calls.

### Ethics, consent and permissions

The participants were recruited by virtue of their professional role, therefore ethical committee review was not required. When recruiting within Uppsala County, approval of responsible supervisors was collected before the social workers were contacted at the request of senior personnel within the County. To ensure that the respondents made an informed decision to participate, they received written information by email. The responsible researcher then offered the possibility to pose questions. If the social workers wished to participate in the study after receiving the information, their written informed consent was collected.

### Data collection

Data were collected between March and May 2020 through individual interviews conducted by telephone. The duration of the interviews were approximately 7 to 20 min, with an average of 15 min. The research was conducted through a qualitative explorative study. Qualitative research was chosen due to its eligibility to study people’s experiences and perspectives [39]. Semi-structured individual telephone interviews were used. The interviews consisted of open-ended questions on different themes. An interview guide was developed (see Table 1) by guidance of King and Horrocks [40], with open-ended questions on four central themes underpinned by the ecological theory [36];

i. social worker’s personal perspective
ii. the influence of the workplace
iii. the interrelation between social workers and Hälsofam
iv. outside organisation influence

**Table 1:** Interview guide

What is your experience of Hälsofam as a working model? Positive/negative	
What do you experience the purpose of Hälsofam to be?	
How do you experience that Hälsofam has succeeded in achieving this purpose?	
How do you think your colleagues experienced Hälsofam?	
How do you think you are affected by your colleagues’ experiences of Hälsofam?	
Is there anything else that can affect your experience of Hälsofam? Rules/policies?	
How has the collaboration between the Social Services and the Hälsofam team worked?	
Concluding remarks, is there anything more you would like to add?	

Due to the situation of the Covid-19 virus, it was not preferable to perform the interviews as face-to-face meetings. The interviews were therefore conducted by telephone. All interviews were recorded with two digital voice recorders, with given consent of all the participants.

### Data analysis

**Fig. 2:**
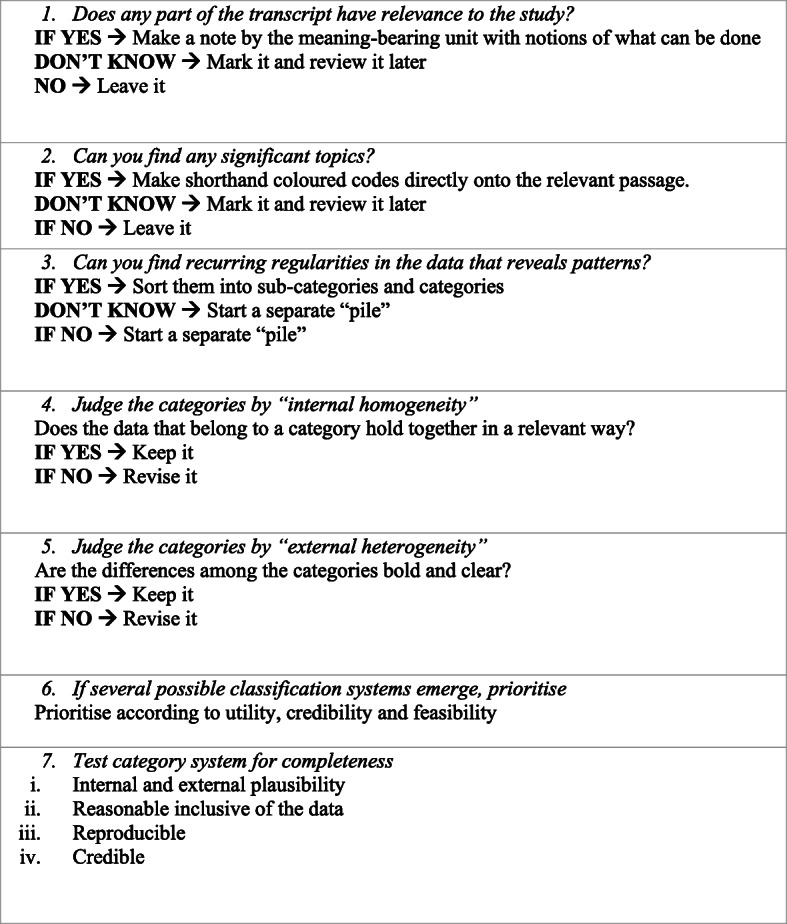
Qualitative Analysis and Interpretation. Based on “Qualitative Research & Evaluation Methods” by M.Q. Patton, 2015, Sage Publications Inc.

A systematic qualitative content analysis with an inductive approach was used to analyse the data [38]. The data were verbatim interview transcripts, and the research used a transcript-based analysis, with supplemented notes from the research protocol. The framework objective was to identify important central factors to understand how the participants view the topic. The analysis process started with several read throughs of all the transcripts, to get a sense of the work. Meaning-bearing units was identified, colour-coded and compared within and between manuscripts. Based on similarities and differences, they were sorted into subcategories and categorised (see Table 2). According to guidance by Patton [38], a procedural data analysis model was developed (see Fig. 2). The first author conducted this procedure along with the second author. The third author reviewed codes, subcategories and categories. This process was made to obtain a trustworthiness within the work.

**Table 2:** Example of the coding process

Text passage	Code	Sub-category	Category
When we are there [at Hälsofam], we are also present when the youth arrives together with their parent or their residential care home. Then we are present a short time before the actual health examination begins. And it feels important that they see, the youth sees, that we’re there. The collaboration becomes clearer. It becomes clear that we are present.	Participation	An interest in participation	For every child and adolescent?

## Results

### Characteristics of participants

A total of 10 social workers took part in the study, 9 within the Uppsala Region and 1 outside of the Uppsala Region. With a degree of Bachelor of Science in Social Work, there are many possible work positions [41]. Of the participants in this study, there were 2 investigators, 7 social secretaries and 1 co-ordinator (see Table 3). Amongst other things, an investigator works with investigating cases where there are suspicions of maltreatment of a child in the home. Within the position of a social secretary, there are work tasks such as placement of children and adolescents in different forms of OHC and provision of support to families with children. The tasks of a co-ordinator are focused on planning and arranging services for children and adolescents in OHC. However, the different positions are to the vast majority similar in their tasks [41].

**Table 3:** Characteristics of the Participants

Variable	Participants
Age (years)	28–63
Gender	8 females, 2 males
Workplace municipality	Within Uppsala County: 9 respondents Outside Uppsala County: 1 respondent
Position within the social service	Investigator: 2 respondents Social Secretary: 7 respondents Co-ordinator: 1 respondent
Years as a social worker	5–29 Median: 11 years
Years with experience of Hälsofam	2–8 Median: 3 years
Caseload age range	Children only: 1 respondent Adolescents only: 5 respondents Full age range: 4 respondents

### Main categories

Three main categories emerged from the analysis. The main categories and sub-categories are presented in Table 4.

- A way in - captures the social workers’ experiences of finding a way into the health care service for the children and adolescents on their caseload. See Table 5.
- Collaboration in action – captures the working relationship between the social service and Hälsofam, and other related services. See Table 6.
- For every child and adolescents? – describes the factors that appear to affect participation in the Hälsofam model. See Table 7.

**Table 4:** Main categories and sub-categories from the data analysis

Sub-category	Category
*The first contact*	*A way in*
*An end to calling around*	
*An ongoing personal relationship*	*Collaboration in action*
*A committed medical team*	
*Working across the silos*	
*Rights and responsibilities*	
*An interest in participation*	*For every child and adolescent?*
*The high workload*

**Table 5:** Main category – A way in

The first contact	*There is a great gratitude that we found [Hälsofam], we found it by chance. We Googled with deep creases in the forehead and were quite irresolute, what should we do?*
	*There have been times over the years that [the Hälsofam personnel] have come and informed a little. Or they have delivered some small quick reference guide on how to perform placements and that say, “Do not forget The Child Protection Team and medical examination”.*
	*If you go in and do a Google search on the Child Protection Team, it comes up. And there are some parents that have... when we have referred to the Child Protection Team, they have Googled it and felt like “That doesn’t seem so good and … then they will take my kids away from me right away” and things like that. So that is a little odd thing which has occurred in several cases actually. Parents Google search.*
An end to calling around	*It’s very nice to have them to turn to in the first place. Then I’ve experienced situations when we have been in a hurry and that they did not have any available appointments, but... It is still nice to have them as an alternative because, otherwise we have to sit and call around to various health centres and doctors at random, so it is nice to be able to turn to them in the first place.*
	*It is an incredible difference compared to the Child Psychiatry Team, where it is more like a closed fort. Impossible to get into.*

**Table 6:** Main category – Collaboration in action

An ongoing personal relationship	*I feel I’ve got a strong relationship with those who work on the Child Protection Team, because they’re not that many and it’s not that high turnover.*
	*The good thing is that they have a staff turnover within The Child Protection Team which is sustainable over time. Basically, we have met the same people since I started working here and we established a contact with The Child Protection Team, until this day.*
	*It is a nice environment when you arrive. The staff are very nice, both at the reception and when you call them. They really make you feel, both me as a social worker and the children and adolescents, to feel seen. They listen very well. They are accommodating there, which I think is very important. That the children and adolescents, when we arrive with them, they feel comfortable and that they do not feel that it is difficult.*
Working across the silos	*The purpose is to get a holistic assessment like that you see... all the different perspectives of a child, both physically and mentally. But also, what we bring with us, knowledge of the family and social difficulties and so on. So that they are assessing health on the basis of every possible … in all perspectives as well.*
	*If I could wish for something and be a little spoiled, or what to say. So, I would like, when we have contact with The Child Protection Team, it is, after all, it is the social service that will requisition all... information. Journals from the Child Welfare Centre and from school and also from public dental care and from other, other county councils and so on. And I think that is... partly it will be like a detour. Because we are going to require all that, and then we will send the material to the Child Protection Team. I could wish for that The Child Protection Team had a holistic perspective on it, so that they were the ones who acquired those records.*
A committed medical team	*One thing that I think is important with the Child Protection Team is that by having the focus that they have on the child, being loyal in that way, they have made it possible, from what I have seen, that they have enabled the children to become more responsive to care. Because many of the children that I meet are very far away from public society and health care. (…*) *And kids who weren’t open at all to psychotherapy and situations when it is hard to just go to a regular doctor. Concerning things like that they have, due to how nurses, doctors and psychologists have treated them, they have become much more responsive to care. I think it has been a very positive experience for them to have doctors, nurses, psychologists who have an understanding for them as children in this situation. And that they can meet them, with that knowledge.*
	*They do not let go like “oh well, our job here is done” and then they let go, but they make follow-ups and makes sure that the youth really get what they need.*
Rights and responsibilities	*I think it’s also a way to protect them, the most vulnerable children in the society, to make sure they get the help and support they need.*
	*To meet the responsibility you take as a society. So to say, when you grant a placement or care for a child.*
	*It is for the legal security of the children. That they should be... their health status when entering social care, it should be investigated. What illnesses they have, if they have been hurt by anything or also in terms of care, that it should be investigated, in what way it has occurred. And mental health problems too, that since children who see violence or are subjected to violence it is also... an issue for The Child Protection Team to help us. For the child to receive support, later, and then as said the legal security as well. For both the authority and the child. And the family.*

**Table 7:** Main category – For every child and adolescents?

An interest in participation	*I usually prioritise being there. I have heard from several colleagues that they sometimes are present over the phone. And, it might make a little difference. Because I think that you get together, that you observe, that creates another dialogue, another meeting, between us and the professionals.*
	*It is clear from our side what to do in this part. Then, like, we let go and hand over to them.*
The high workload	*Due to our workload, sometimes we might have to prioritise things that can be done in an easier way.*
	*What happens is that the children get there on a medical check-up, and maybe a psychology screening. And so the youth in my case says lots of things like that “yes but I feel pain in my knee” or whatever and then the CPT make a referral to the health care in the region (…) me as a referral investigator is then expected to be in the heart of things, because then they send a care summon if they send an internal referral to.. well some physiotherapist or whatever we confirmed at this health examination. And then it is assumed that it is me as a referral investigator who will convey this appointment. Not the legal guardians, which can… feel a bit cumbersome, when you are a referral investigator.*
	*It works well, it is just the small things that do not... I mean, of course it is difficult to book appointments whenever you need one.*

#### The first contact

*“The first contact”* is based on how the social workers talked about the perceptions of the first contact with Hälsofam. For social workers, those outside of Uppsala County had their first contact with Hälsofam through a Google Search. They had desperately been searching for a solution to their problem gaining access to health care for their cases and expressed great gratitude for finding Hälsofam. Other social workers within Uppsala County were introduced by colleagues or received information directly from the team. Being introduced by a colleague was perceived positively. Whereas some experienced the information about Hälsofam via a standard informational routine, which aimed to remind the social workers to use the team and how to use them.

In terms of parents and legal guardians’ first contact, one respondent described several scenarios when they had been searching for information on Hälsofam online and were affected by negative publicity and a perceived ‘bad reputation’. That first contact resulted in worried parents, aroused suspicion and fear of what could happen if their child came in contact with Hälsofam. The parents’ first contact had affected the respondent’s work negatively.

#### An end to calling around

*“An end to calling around”* is based on the previous experiences of the social workers, being forced to call around to doctors and health care centres, trying to book appointments for children and adolescents. Several respondents shared their previous experiences of the time-consuming process of phoning doctors and health care centres, having their calls being put on hold and not having the possibility or knowledge to get in contact with the right actor. Their perception of Hälsofam was that now they had a direct, efficient contact point. By being given access to the Hälsofam network and their connections, they experienced that they got the right support faster. In cases where the Hälsofam personnel could not help them directly, they experienced that the team could help them with referrals to other health care actors.

#### An ongoing personal relationship

When considering collaboration and coordination, the low staff turnover within Hälsofam is discussed by several respondents. The experience that the social workers have been able to create and maintain a personal relationship with the team is appreciated.

Those who reported that they had a close contact with the team, emphasised that the work was perceived easier with a personal relationship. They described the experience of having met the same people from day one of their work and the comfort and support that brought.

Some respondents also highlighted the positive effects on the children and adolescents, and other actors within the process, who witnessed this relationship. They perceived it being positive that others could experience their good relationship, creating a sense that the social service and the Hälsofam personnel worked together for the child or adolescent.

#### Working across the silos

All the respondents had the perception that Hälsofam worked with another point of view than the social service and other actors within the health care sector. Previous experiences have been that other actors only focus on their specialism. For example, Child Psychiatry Teams focused only on mental health, and Child Health Centres only focused on physical health. Hälsofam, on the other hand, have enabled assessments that include mental, dental and physical health as well as seeing to the context around the child or adolescent. At the question of the purpose of the Hälsofam model, the social workers witnessed an understanding of possible parental behaviour in the kinds of situations experienced by children in OHC, as well as knowledge of social inequalities and potential exposure to violence and threats. Being sensitive to other relevant perspectives, other than just “your blood tests look okay and you have a normal height”, created a more holistic point of view of a child’s or adolescent’s health status. The majority described this as something unusual, compared to other actors within the field.

The social workers described their perceived limitation within the field of health care and lack of medical knowledge. Several of the respondents had the experience that Hälsofam had been of valuable help in the work of mapping the health status and medical history of children and adolescents. The specialist expertise of the Hälsofam personnel, beyond that of social workers, was clearly evident to the participants, with several speaking to the value of having a trauma psychologist connected to the team. In some cases, children and adolescents could have been experiencing several placements in different municipalities and sometimes other regions, making it difficult for the social worker to trace the medical history. Some respondents experienced that the Hälsofam personnel had then been able to assist the work in finding medical records from a young age. Yet, others wished for an even greater holistic approach within the medical field and the requisition of relevant information.

#### A committed medical team

All 10 of the respondents agreed on the perception that the Hälsofam personnel were a committed medical team. They argued that the team at its core were loyal, sensitive and dedicated in their work towards a better health for children and adolescents in OHC. With this focus, some experienced that “their” children and adolescents had developed a more trusting and responsive approach to the health care sector.

Several of the respondents spoke about previous experiences of children and adolescents “falling through the cracks” in the work process of other institutions. Their experiences were that the Hälsofam personnel have been dedicated and eager to ensure that they fulfil their responsibility and provide the child or adolescent with necessary care. They experienced that the personnel were being committed and willing to go that extra mile for the children and adolescents. Some social workers reported a sense of the team being generous with their knowledge and time, and that the team “wants to be there, they want to help you and the child”.

#### Rights and responsibilities

Several respondents discussed the rights of the child and the different responsibilities of various actors, to ensure those rights. Some respondents perceived that the Hälsofam personnel ensured that the different authorities and legal guardians met their responsibilities towards the child. They had the experience that Hälsofam framed and coordinated the different responsibilities, so that everyone worked together for the sake of the child.

The respondents argued differently, where some perceived that it was a shared societal responsibility to care for the child and adolescent. Others argued that actors should work separately and meet the responsibility within their profession.

One respondent had the experience that Hälsofam personnel highlighted, framed and ensured the legal security for the child and adolescent, for the legal guardian, for the residential care homes and for the social service. The respondent argued that it was essential to know the medical history of a child from the very beginning of the social care, to ensure the right actor is to hold responsible for any potential injury, sickness or ill-health. Several respondents also argued that the Hälsofam personnel more clearly stated the responsibilities according to Swedish legislation.

#### An interest in participation

The level of participation of the individual social worker was something that the respondents discussed in different ways. Some respondents considered it important to attend the health assessment process, and therefore prioritised it. They had the perception that they were included and welcomed into the process, with a positive feeling that the Hälsofam personnel and the social service had a shared responsibility throughout the process and that the child or adolescent were “their common child/adolescent”. By being present they experienced a deeper understanding of how the social service could provide better support for the child or adolescent, and that meetings in person enabled better dialogues between the actors.

Other respondents did not, to the same extent, report the same interest of participation. They were present over the telephone and did not express a wishing of being present in person. Some respondents perceived that they got the relevant information they needed from verbal and digital communication.

The respondents that expressed an interest in active participation reported a higher usage of the team. They consider it being important to refer children and adolescents in the process of OHC to Hälsofam. On the other hand, the respondents that expressed a lower level of interest in participation and attendance, reported to a higher extent that they used Hälsofam “sometimes”, “for some children” and/or “whenever they remembered to have them in mind”. This finding highlighted an inequality, that an interest in participation could have an impact on whether or not a child or adolescent received care from Hälsofam.

#### The high workload

The high workload within the field of social work affected the actor’s ability to perform equivalent work efforts for every child or adolescent. The high workload was prominent both within the social service and in Hälsofam. The social service as an authority experienced a high workload, forcing the social workers to sometimes choose the ‘easiest way’. Taking meetings and briefings over the telephone were given as an example of this. The social workers’ ability to be present throughout a process could therefore be due to the workload and/or the work routines within the profession.

The process of referring to and working with Hälsofam was perceived as time consuming for some respondents. The fact that different professions within the social service had different workloads and working conditions, was raised by some respondents as a somewhat ‘unfair’ situation. Being a referral investigator or case worker could in some cases affect the prerequisites to participate and have an active attendance for every child and adolescent. Referral investigators tended to work a heavier caseload and thus have less capacity per child, whereas a caseworker had more time to dedicate to each child on their caseload. Some participants expressed that Hälsofam actually added to their workload, having the perception that they might see too much to the child’s every expressed need. They would argue that it resulted in a lot of extra work for the social worker.

The workload in the health care sector was also an argument for the inability to work for every child or adolescent. Some respondents could experience difficulties in being able to book appointments at Hälsofam when they needed to, with an argument that “it is the situation of the society today”.

## Discussion

The purpose of this research was to study how social workers perceive and experience the Hälsofam model. In the problem explanation, it was stated that a well-functioning system of health assessments and examinations is necessary to discover potential health issues concerning children and adolescents in OHC. Basic preventive needs have been unmet and there has been a need for a comprehensive and continuous system.

The respondents’ perceptions and experiences have, from different perspectives, described to what extent Hälsofam has worked as a well-functioning system. The first impression was prominent in participants’ responses. The *microlevel* of the ecological model can be used to understand the findings in the perceived first contact with Hälsofam [36]. Looking to existing literature regarding initial service contact, lack of appropriate information and stigma have been identified as operational barriers to engaging young people in OHC and their carers in health services [24]. Previous research also indicates that parents feel an overwhelming sense of fear during their interactions with child protective services, and this fear is most pronounced at the time of the first visit [42]. It is therefore understandable that social workers emphasised the first impression of Hälsofam among clients as important.

The Swedish health care system has been under pressure since the 1990s, following increased critical observation and demand for cost-containment [43]. Although the system aims to provide equitable health care [44], research has shown that this is not being achieved and socioeconomic factors appear to be related to health care utilisation, including among young people [45]. Thus, the health care sector access issue described by the social workers in the present study, albeit an inter-agency access issue rather than an individual-level access issue, was somewhat expected. The reports of Hälsofam overcoming this issue aligns with the logic model for the initiative and is suggestive that Hälsofam is contributing to greater health equity. The positive effects of the finding “a way in” is in line with the *mesosystem* in Bronfenbrenner’s ecological model [36]. This ‘way in’ to the system can lead to preventative action whereby health concerns are detected earlier and the need for more extensive health care utilisation at a later stage is mitigated. This is in line with previous assessment of the societal costs of health and dental care organisational models for children and young people in OHC [22].

The social workers’ perception of Hälsofam was strongly connected to the collaborative nature of the work. Relationships and the sense of commitment were very important to some of the respondents, creating a deeper meaning of the collaboration than just performed tasks. Personal relationships and commitment within the medical team, are in line with the structure of the *mesosystem* [36]. Positive attitudes towards other professionals has been shown to be an important factor in cross-agency collaborations [46, 47], with increased familiarity appearing to foster positivity [46, 48, 49]. Related to this, the present study uncovered social workers’ appreciation for the low staff turnover in the Hälsofam team. Whether this attribute of staff retention can be credited to the Hälsofam model and replicated in other areas upon scale-up is not known. Yet, it validates the understanding that familiarity among multidisciplinary team members is important and should be encouraged by the Hälsofam leadership team.

Some participants emphasised the relevance of high standards within the work performance, the ability to work inter-professionally, and responsibility towards the child or adolescent. This aligns with previous research findings that emphasise professional skills and knowledge as key to cross-agency working [31]. For example, in a survey of mental health and child protection services personnel in Queensland, Australia professional knowledge was reported as crucial to collaboration [50]. That the social workers in the present study lifted the importance of the ability to work inter-professionally also supports previous research findings. A survey of child welfare and substance abuse treatment workers in the United States found that those who reported skills and knowledge in collaboration were more likely to collaborate with personnel from other professions [47]. Responsibility towards the child or adolescent consisted of mixed views among the participants in the present study, which is reflective of the wider field of inter-agency working; differing philosophies are commonly cited as an issue [31] and the importance of reconciling perspectives ahead of working together has be emphasised [51, 52]. That Hälsofam was described as taking an active role in framing and co-ordinating the different responsibilities of the professionals involved is an encouraging finding, and aligns with the Bronfenbrenner *exosystem* [36]*.*

Despite the improved work experiences and the positive perceptions, there appeared to be a form of selection bias with regard to which children and adolescents were referred. This could be interpreted as the model not achieving the goal to fulfil the basic needs of *every* child or adolescent in OHC. An inequality emerged from the interviews, showing that the usage of the Hälsofam care depended on the personal interest and the experienced workload of the individual social worker. This can be understood from Bronfenbrenner’s definition of the *person* [36]. Beyond the aforementioned work-related philosophies and attitudes towards other professionals, the personal interest of social workers does not appear to have been explored in relation to inter-agency working. The present study uncovered mixed responses in terms of the level of interest in Hälsofam, but the mechanism behind this is unclear and warrants further investigation. On the other hand, it is widely reported that social workers experience high caseloads and work-related stress, more so than comparable occupational groups [53]. In connection to cross-agency working, finding the time for collaborative work has been highlighted as a barrier in several studies [48, 54, 55]. Therefore, although it contradicts the logic model for Hälsofam in which equitable access is fundamental, the connection between social worker workload and Hälsofam usage is not surprising, due to the societal setting in the *exosystem* [36]. The new national regulations on health assessments [18], implemented only a couple of months prior to the interviews being conducted, have potential to positively affect access to Hälsofam. Prompts on policy impact were included in the interview question guide for the present study in an attempt elucidate social workers’ perceptions on these regulations yet no findings on the topic emerged. This could be due to the very recent implementation of the regulations and the time lag sometimes seen in policy affecting practice. One could also question whether the necessary attention has been given at the strategic level, which can affect how well organisational policies function [24].

The findings of this study emphasised the relevance of positive interrelations to succeed with co-ordination and collaboration. The limitations, based on the research findings, showed that potential inequalities could occur when decisions of Hälsofam usage were linked to the social workers’ personal views and decisions. Other limitations could come from the societal situation in which the social service and Hälsofam operate. Societal responsibility, and the resulting heavy workload, affects to what extent the social worker is able to work for every child or adolescent in the same way. This creates a somewhat unjust situation for the child or adolescent, being dependent on the personal views of the social worker and the situational workload at the moment.

### Strengths and limitations

Strengths of this research was having several people involved in the data analysis and analysing the research findings according to a pre-chosen theoretical framework. By that, the reconstruction and representation of the social workers’ narratives were according to the reality of the ecological theoretical framework and not according to the personal interpretation of the responsible researcher. By using the method of verbatim interview transcripts, an interview guide and an interview protocol, the research limited irrelevant interpretation of the data. The interpretations of the findings were confirmed by using quotations from the verbatim interview transcripts. To ensure a logical, traceable and documented working process the research was made by describing the research design thoroughly, including the planning, recruitment, data collection and analysis process.

Potential limitations of this paper were since the aim of the current research was to study the experiences and perceptions of social workers, specifically concerning the Hälsofam model, it was a very narrow field. Given the reliance on only the social worker perspective, the application of the ecological model was somewhat limited; however, the application of the model was intended to broaden the scope of the social workers’ responses to consider the various systems in which Hälsofam can be interpreted.

Another potential limitation was the sampling method. Due to the situation of the Covid-19 virus, the studied group of professionals were difficult to establish contact with within the timeframe of the research. Therefore, a mix of purposeful sampling and snowballing sampling was conducted. As Hälsofam representatives initiated the purposeful sampling, there is a risk of biased perspectives whereby those who would give a positive interpretation of the model were selected. Snowball sampling also involves the limitation of not being able to provide a representative sample, because of the risk to only reach respondents with similar point of views. The lack of representation of the municipalities could also be considered a limitation. Within Uppsala County, there are 8 municipalities. All 8 have an established collaboration with Hälsofam. In the current research, two municipalities within Uppsala County and one within Stockholm County (included in service provision given the proximity to Uppsala County) were represented. A further limitation was the short length of the interviews, which could be a result of the interview guide being too narrow, or too few probes and follow-up questions [38]. A common way of using elaboration probes is nonverbal, gently nodding the head as a positive reinforcement. An obstacle for that in the current research was the absence of face-to-face conversations. For the interviewer, it was a limitation not being able to see the facial expressions and body languages of the respondents. Further to this, the data collection process depended on the social workers’ availability. The contacted social worker could call up the responsible researcher whenever they had the possibility, which may have led to a lower level of researcher preparedness. Moreover, social workers may have kept the conversation relatively brief due to competing demands and the high workload that they speak of in the interviews.

## Conclusion

The findings of this study emphasise the importance of the organisation for health assessments of children and adolescents in OHC. The collaboration between the social service and Hälsofam has several reported strengths. A perception of a strengthened organisation and work force, and an improved working process concerning availability, competence and effectiveness are all positive improvements that were reported. The limitations of the Hälsofam model uncovered by this study point to the importance of creating routines and structures for usage of the Hälsofam model, as well as the relevance of more support and resources to this societal activity. An important direction for future research could be to examine experiences and attitudes of others connected to, and involved in, Hälsofam. Interviews children and adolescents with experiences of being referred to Hälsofam would give valuable insights on how the collaboration and work process have been perceived by those in need of care. To examine the attitudes of parents and caregivers, and other actors involved in the Hälsofam work, would be useful to support the future well-being of Hälsofam users.

## Supplementary Information

**Additional file 1.**

## Data Availability

The data that support the findings of this study are available on request from the corresponding author. The data are not publicity available due to containing information that could compromise the privacy of research participants.
