# Supplementary material for: Social workers’ perspectives on a medical home model for children and adolescents in out of home care – an interview study
Source: BMC Health Serv Res. 2021 Aug 12;21:804. doi: 10.1186/s12913-021-06737-1 (PMC8359767; doi:10.1186/s12913-021-06737-1)
Supplement: Supplementary file 1 — Additional file 1. [file 12913_2021_6737_MOESM1_ESM.docx]

Social workers’ perspectives on a medical home model for children and adolescents in out of home care – An interview study

Nina Johansson, Child Health and Parenting (CHAP), Department of Public Health and Caring Sciences, Uppsala University, Uppsala, Sweden, [nina.johansson@pubcare.uu.se](mailto:nina.johansson@pubcare.uu.se)

Karin Fängström, Child Health and Parenting (CHAP), Department of Public Health and Caring Sciences, Uppsala University, Uppsala, Sweden, [karin.fangstrom@pubcare.uu.se](mailto:karin.fangstrom@pubcare.uu.se)

Georgina Warner, Child Health and Parenting (CHAP), Department of Public Health and Caring Sciences, Uppsala University, Uppsala, Sweden, [georgina.warner@pubcare.uu.se](mailto:georgina.warner@pubcare.uu.se)

Interview guide

1. What is your experience of Hälsofam as a working model?

- Positive/negative?

1. According to your perception, what is the purpose of Hälsofam?
2. According to your perception, how well have Hälsofam succeeded in achieving that purpose?
3. From your point of view, how has your colleagues experienced Hälsofam?
4. How do you believe you are affected by your colleague’s experiences of Hälsofam?
5. Is there anything else that can affect your perception of Hälsofam?
6. How has the collaboration between the Social Service and the Hälsofam team worked?
7. Concluding remarks, is there anything else you would like to add?
